# Beneficial effects of Fu-Zheng-Qu-Zhuo oral liquid combined with standard integrated therapy in patients with chronic kidney disease (stage 3–4): A randomized placebo-controlled clinical trial

**DOI:** 10.1097/MD.0000000000007448

**Published:** 2017-07-14

**Authors:** Shen Li, Xiang-Rong Rao, Xi-Wen Dai, Kun Pei, Lee Wang, Bao-Min Huo, Xiu-Juan Wang, Ling-Xin Kong, Nan-Nan Zhang, Feng-Mei Lian

**Affiliations:** a Nephrology Department of Guang’anmen Hospital, China Academy of Chinese Medical Sciences; b Nephrology Department of South Area of Guang’anmen Hospital, China Academy of Chinese Medical Sciences; c Nephrology Department of Beijing Fangshan Hospital of Traditional Chinese Medicine; d Drug Clinical Trial Institution of State Food and Drug Administration of Guang’anmen Hospital, China Academy of Chinese Medical Sciences, Beijing, China.

**Keywords:** chronic kidney disease (stage 3–4), complementary therapies, eGFR-Slope, Fu-Zheng-Qu-Zhuo oral liquid, randomized placebo-controlled clinical trial

## Abstract

**Background:**

The high worldwide prevalence of chronic kidney disease (CKD) is a critical health problem and the development of more effective therapies is urgently needed. We conducted a randomized, double-blinded, placebo-controlled clinical trial from October 2010 to December 2012 to assess whether Fu-Zheng-Qu-Zhuo oral liquid (FZQZ) has a beneficial effect in preventing CKD progression when added to standard integrated therapies.

**Methods:**

Patients with CKD stage 3 to 4 from 3 hospitals in Beijing, China were enrolled. Patients were randomly assigned to the FZQZ or placebo groups and were treated with standard integrated therapy plus FZQZ or placebo (20 mL each time, 3 times/d) for 12 months. Patients received post-trial follow-up until October 2014. The primary outcome was the estimated glomerular filtration rate (eGFR)-Slope (mL/min per 1.73 m2 per month) during the in-trial time, which was calculated by the eGFR regression curve estimated from each serum creatinine measurement during the in-trial period. Secondary outcomes were changes in 24-h urine protein excretion (24-h UP) and albumin and hemoglobin levels from baseline during the in-trial period. Time to composite endpoint events (initiation of long-term dialysis, doubling of serum creatinine, or CKD-related death during the in-trial and post-trial phases) was assessed as a secondary outcome.

**Results:**

A total of 68 patients (43 in the FZQZ group and 25 in the placebo group) completed the in-trial and post-trial phases, with an average follow-up time of 31.6 ± 9.6months. The FZQZ group had amean eGFR-Slope of 0.25 ± 1.44 as compared with −0.72 ± 1.46 (mL/min per 1.73m2 per month) in the placebo group during the in-trial period (*P* = .003). The FZQZ group showed decreased 24-h UP, with a change from baseline of −0.08 (interquartile range [IQR], −0.33 to 0.01) versus 0.01 (IQR, −0.19 to 0.33) g/24h in the placebo group (*P* = .049). Decreased risk of composite endpoint events was observed only in the post-trial phase (hazard ratio = 0.42, 95% confidence interval: 0.16–1.11, *P* = .038). No significant differences in albumin and hemoglobin level changes were observed.

**Conclusion:**

Adding FZQZ oral liquid to standard integrated therapies may aid in attenuating CKD progression.

## Abstract 2

Supplemental Digital Content is available in the text

## 1 Introduction

Chronic kidney disease (CKD), with a worldwide prevalence estimated at 8% to 16% worldwide, is an increasingly critical public health problem with a heavy social economic burden.^[1,2]^ Current integrated intervention strategies to prevent and treat CKD include the following: primary disease control, lifestyle changes (dietary measures, smoking cessation, exercise), use of angiotensin-converting enzyme inhibitors (ACEIs) or angiotensin II type 1 receptor blockers (ARBs), improving kidney anemia, supplementation with bicarbonate to correct metabolic acidosis. However, these strategies, along with other protocol-driven medical therapies to control blood pressure (BP), glucose, and lipid levels in accordance with the Kidney Disease: Improving Global Outcomes (KDIGO) clinical practice guidelines, have only achieved modest results. Therefore, the development of more effective therapies is necessary to help attenuate CKD progression.^[3]^

Traditional Chinese medicine (TCM) has been used to treat kidney diseases for thousands of years in China, and the efficacy of TCM has been widely published and reviewed in recent decades.^[4]^ Fu-Zheng-Qu-Zhuo (FZQZ) oral liquid is a compound herbal medicine formulation. It has been used to treat patients with CKD stage 3 to 5 in clinical practice from the 1990s, and more than 200 cases of its effectiveness have been reported.^[5–7]^ In our previous investigation, FZQZ oral liquid was found to decrease urinary protein excretion (UP), reduce extracellular matrix accumulation, inhibit transform growth factor β over-expression, alleviate glomerulosclerosis, and mitigate tubular-interstitial fibrosis in subtotal nephrectomy rats.^[8,9]^ However, a well-designed clinical trial is required to evaluate the efficacy and safety of FZQZ. Thus, we conducted a multicenter, randomized, double-blinded clinical trial to determine whether the addition of FZQZ oral liquid (60 mL/d) to the standard integrated therapy resulted in further kidney protection in patients with CKD stage 3 to 4.

## 2 Methods

### 2.1 Study design

This multicenter, randomized, double-blinded, placebo-controlled clinical trial was conducted from October 2010 to December 2012 in 3 hospitals in Beijing, China. The protocol was approved by the Research Ethics Committees both at the central coordinating center (Ethics Committee of Guang’anmen Hospital) and at each of the participating sites (central ethics committee approval number: 2010052). The study was conducted in accordance with the ethical principles of the Declaration of Helsinki, the Good Clinical Practice guidelines of the International Conference on Harmonization, and local regulatory requirements. The trial was registered at the US National Institutes of Health (ClinicalTrials.gov) NCT02275468.

### 2.2 Study population

Patients who met the following criteria were included in the study: aged 18 to 75 years; signed informed consent; had an estimated glomerular filtration rate (eGFR) of 15 to 59 mL/min/1.73 m^2^ using the CKD Epidemiology Collaboration equation (CKD-EPI) on at least 2 dates separated by at least 90 days but no more than 6 months; and had not received any other TCM treatment for at least 2 weeks. Patients were excluded if they had any of the following conditions: an immediate need for dialysis; a 50% increase in serum creatinine (SCr) that occurred within 2 months before screening; myocardial infarction or cerebrovascular accident 3 months prior to the trial; connective-tissue disease, obstructive uropathy, or renal transplantation; severe disease in other organs, cancer, psychotic diseases, or active tuberculosis; future or current treatment with corticosteroids and/or other immunosuppressive agents; hemoglobin (HGB) < 80 g/L; diabetes mellitus with uncontrolled blood sugar [glycated hemoglobin (HbA1c) > 8.0%]; systolic BP more than 140 mm Hg or diastolic BP more than 90 mm Hg when combined with the use of 3 kinds of anti-hypertensive agents; women in pregnancy or lactation; and enrollment in other trials.

All patients gave informed consent prior to any study-related procedures.

### 2.3 Study intervention

This study had 3 phases: pre-trial, in-trial, and post-trial. During the 2-week pretrial phase, all of the recruited patients received integrated therapy in accordance with the KDIGO CKD guideline. Details regarding the treatment protocol are included in the Supplementary Information section.

In the 12-month in-trial phase, patients in the FZQZ group received FZQZ oral liquid 20 mL, 3 times a day in addition to the integrated therapy; patients in the placebo control group received the placebo at the same volume and frequency in addition to the integrated therapy. FZQZ oral liquid is composed of 14 Chinese herbs that are listed in Supplementary Table 1. FZQZ oral liquid was prepared by decoction, concentration, ethanol extraction, alcohol recycling, and filtration in the Pharmaceutical Laboratory of Guang’anmen Hospital, Academy of Chinese Medical Science, China (License code: Z20063242). Quality control pertaining to ingredients and bacterial contamination was performed according to good manufacturing practice standards. The placebo formula, also provided by the Pharmaceutical Laboratory of Guang’anmen Hospital, contained melanoidin malt and water, and had the same packaging and appearance as the FZQZ oral liquid.

The post-trial phase was open-labeled and the FZQZ therapy was continue at the patient's discretion; however, the placebo was not provided.

### 2.4 Randomization and blinding process

Eligible patients were randomly allocated in a 1:1 ratio into 2 groups (FZQZ or placebo group) based on a computer-generated randomization schedule list using SAS (version 8.0, SAS Institute Inc., Cary, NC, USA). Before the onset of the study, a unique 4-digit number in ascending numerical sequence was randomly assigned to treatment with either the FZQZ oral liquid or placebo. Investigators and participants were blinded to the participant's treatment throughout the course of the study. A label indicating that the treatment assignment was provided to the investigators in a separate sealed envelope for each participant. The placebo was similar to the FZQZ oral liquid in size, color, and shape, with the absence of the active herbal medicine. Study medicines were blinded, packaged, and provided to an investigation site as a bulk shipment in accordance with a randomization schedule.

### 2.5 Outcome measures

Patients were scheduled to have a screening visit and an initial visit in the pretrial phase, and then monthly follow-up visits during the in-trial and post-trial phases.

After the screening visit, demographic and baseline characteristics of the enrolled patients were collected. Baseline levels of SCr, blood urea nitrogen, albumin (ALB), bicarbonate, potassium, alanine aminotransferase (ALT), HGB, 24-h UP, and HbA1c for diabetes mellitus patients were measured within 7 days after the first dose of the study medicine was given. Baseline eGFR was also calculated in this period according to the CKD-EPI equation.

During the in-trial phase, patients had a clinic visit every month, a blood test to measure the above-mentioned parameters every 2 months, and a 24-h UP test every 6 months. Patients were asked to self-monitor their BP at least twice a day, and patients with BP out of the target range more than 30% of the time were considered uncontrolled. Changes in the symptoms were recorded monthly. The final values of these parameters were collected within 5 days after the last dose of study medicine was given or were collected before the participants dropped out during the in-trial period. Missing eGFR dynamic curve generation and eGFR-Slope calculation data for the participants who dropped out were carried forward using the last parameters that had been collected before the participants dropped out during the in-trial phase.

In the post-trial phase, patients had a clinical visit every month, with a blood test for the above-mentioned parameters and a 24-h UP test every 6 months.

The primary outcome was the decline in eGFR during the in-trial phase (eGFR-Slope, mL/min per 1.73 m^2^ per month). The mean eGFR-Slope for each eligible patient was calculated by the eGFR regression curve, which was estimated from each SCr measurement during the in-trial phase. The secondary outcomes were changes in 24-h UP and ALB and HGB levels from baseline to the end of the in-trial phase. Time to composite end-point events (defined by initiation of long-term dialysis, CKD-related death, or doubling of SCr) during the in-trial and post-trial phases were assessed as a secondary outcome. Patients with kidney failure events (defined by the initiation of long-term dialysis or the doubling of SCr) were removed from the trial. Changes in ALT and potassium from baseline during the in-trial phase were collected as safety parameters. Patients were also asked to report any symptoms or adverse effects at each follow-up visit or immediately as they occurred.

Severe adverse events were defined as all-cause death, emergent or fatal incidence of severe infection, potassemia, liver injury, or cardiovascular incidence that required hospitalization.

### 2.6 Sample size and statistical analysis

According to previous reports, the GFR decline in “fast progressors” was >4 mL/min per 1.73 m^2^ per year (≈−0.33 mL/min per 1.73 m^2^ per month),^[10]^ and our preliminary work showed the mean GFR decline in patients with CKD who were treated with FZQZ was 0.06 mL/min per 1.73 m^2^ per month,^[11]^ with a standard deviation (SD) of 0.48, a power of 80%, and a significance level of 5%. Thus, a total of 60 patients would be needed in each group after accounting for a dropout rate of 15%.

Normally distributed quantitative data were expressed as mean ± SD and compared using the *t* test or analysis of variance. Nonparametric variables were expressed as median and interquartile range (IQR) and compared using the Mann–Whitney *U* test. Categorical data were analyzed using the chi-squared test across the FZQZ and placebo groups. Since the majority of the patients had primary glomerular disease (PGD), and patients with diabetic nephropathy (DN) with overt proteinuria experience rapid declines in GFR,^[12]^ a subgroup analysis according to CKD-cause (PGD, DN, and others) was performed. Kaplan–Meier methods were used to assess the secondary outcome of time to composite end-point events and their significance was assessed using the log-rank test. The Cox proportional hazards model was used to evaluate the hazard ratio (HR) of the kidney failure events during the entire follow-up period with respect to the treatment with the FZQZ oral liquid in the in-trial phase. Treatment with FZQZ, sex, age, CKD-cause, baseline eGFR, ALB, HGB and 24-h UP levels, comorbidity of diabetes mellitus, target BP that was achieved, and combined treatment with ACEI or ARB were contained in the multivariate Cox regression model. The model was built up without selection processes. The primary and secondary outcomes were analyzed according to the intention-to-treat (ITT) principle. The data of patients who had taken at least 1 dose of the study medicine with at least 3 time intervals of measurements taken (baseline, final, and one in-trial phase test) were all included in the ITT analysis. All *P* values were 2-tailed; *P* < .05 was considered statistically significant. Data were analyzed using SPSS 11.0 (SPSS Inc., Chicago, IL).

## 3 Results

### 3.1 Study population and compliance with the study medicine

**Figure 1:**
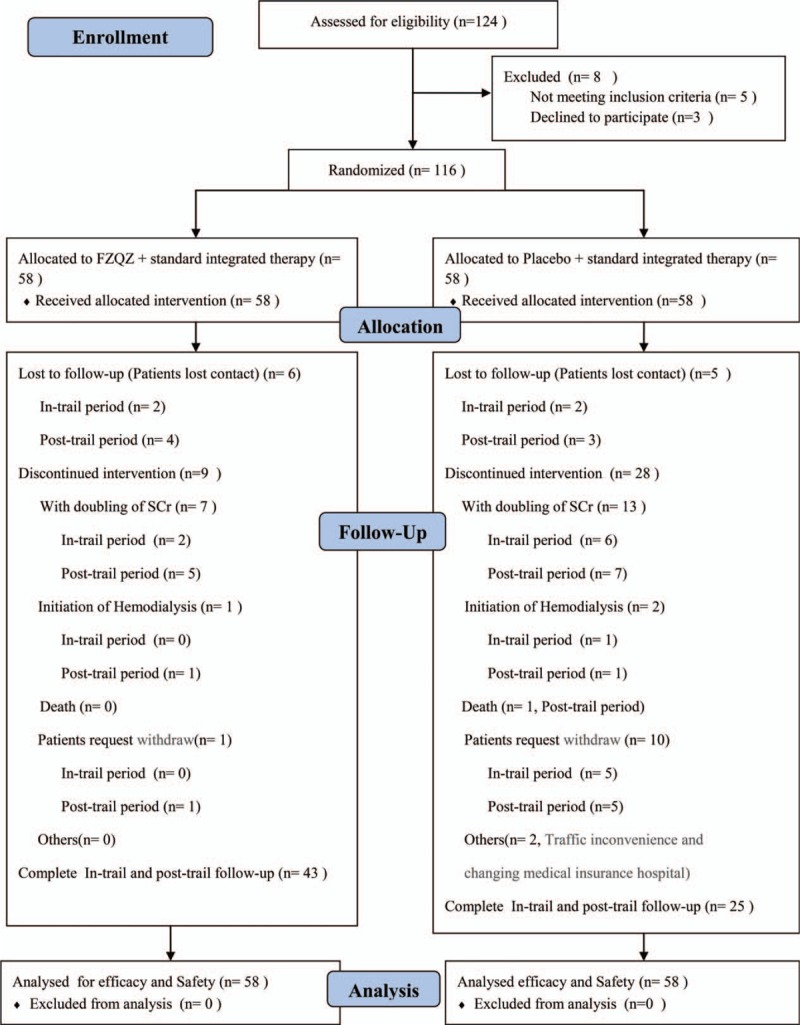
Randomization and participant flow of patients with CKD stage 3 to 4 who were enrolled in the study. CKD = chronic kidney disease, n = number of patients in the respective population, SCr = serum creatinine, discontinued intervention = withdrawn by patients or doctors.

A total of 124 patients were recruited for this study, 8 of which were excluded based on the exclusion criteria. The remaining 116 patients were randomly assigned to the FZQZ group or the placebo group (58 patients in each group) from October 2010 to December 2012. The post-trial follow-up ended in October 2014 due to a grant limitation. There were 11 dropped-out participants (2 in the FZQZ group and 9 in the placebo group) at the end of the in-trial phase, and another 13 dropped-out participants (5 in the FZQZ group and 8 in the placebo group) in the post-trial follow-up phase (patient disposition and the details pertaining to the dropouts are shown in Fig. 1). The final study visit occurred in October 2014, and the average follow-up period was 31.6 ± 9.6 months. Although medicine compliance was high in both groups, the compliance rate was lower in the placebo group than in the FZQZ group (86.2 ± 20.0% vs. 94.1 ± 15.6% [*P* = .020], respectively). The method used to capture patient compliance and the analysis of the compliance rate is shown in Supplementary Table 2.

### 3.2 Demographic and baseline characteristics of enrolled patients and the main combined therapies

The demographic and baseline characteristics of the enrolled patients were similar between the 2 groups (Table 1). Among the 50 patients with PGD, 29 had biopsy-proven diagnosis, including 21 with IgA nephropathy, 3 with mesangial proliferation glomerulonephritis, 3 with idiopathic membranous nephropathy, 1 with focal segmental glomerulosclerosis, and 1 with minimal-change disease. The other 21 patients with PGD were clinically diagnosed. Eight out of the 14 patients with DN had biopsy-proven advanced diabetic glomerulosclerosis, while the other 6 patients were clinically diagnosed. Ischemic nephropathy referred to atherosclerotic renal artery stenosis, which was clinically diagnosed by renal artery imaging.

**Table 1:**
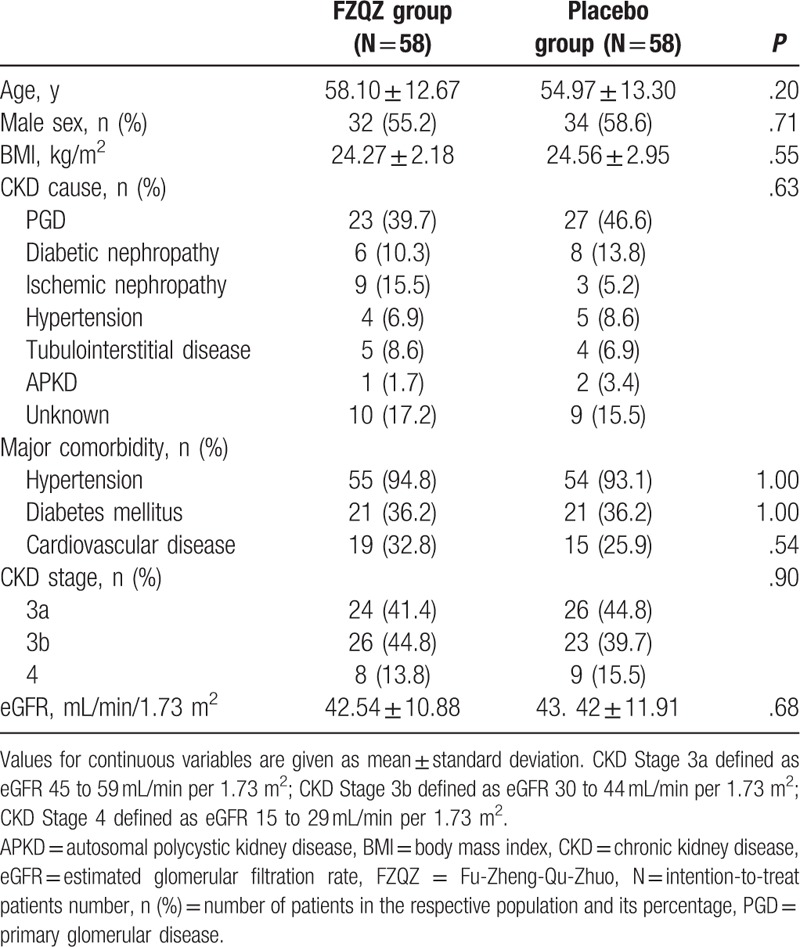
Demographic and baseline characteristics of the patients after randomization.

The main combined therapies during the in-trial phase were compared (Table 2) and no statistically significant differences were observed.

**Table 2:**
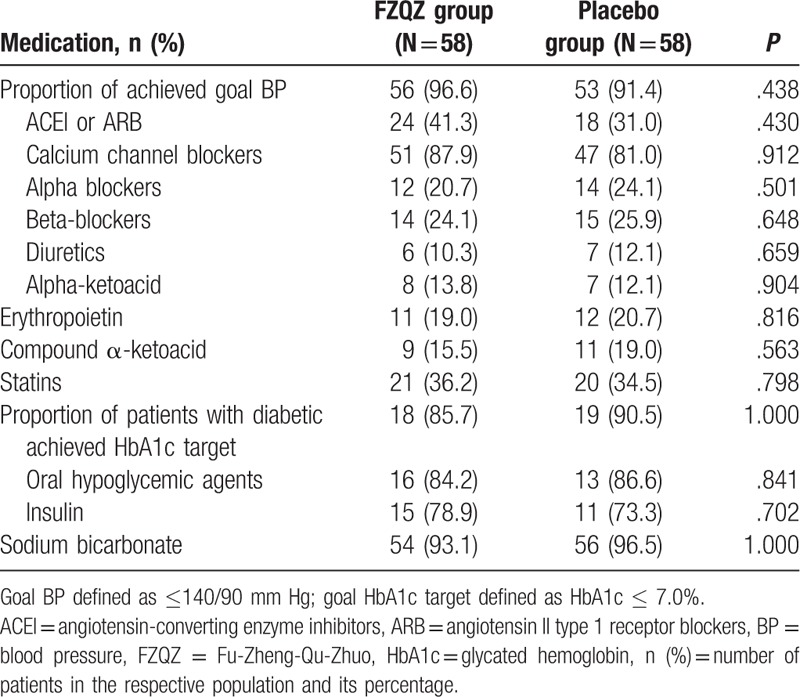
The main combined therapies during the in-trail phase.

### 3.3 Primary outcome

**Figure 2:**
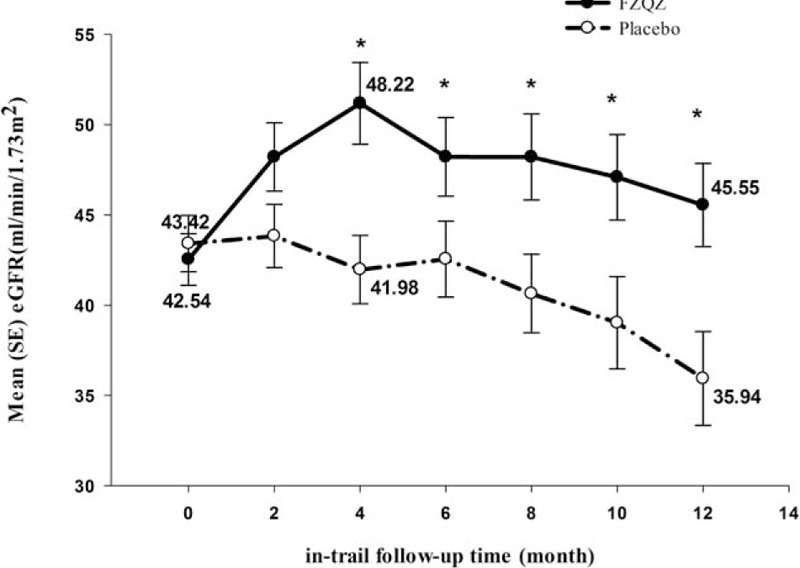
The eGFR trajectories of the FZQZ and placebo groups in the in-trial phase. Compared with the placebo group, eGFR was stable and somewhat increased in the FZQZ group, and there were significant differences between the FZQZ and placebo groups in terms of eGFR from time-point month 4. Data presented as mean ± standard error. Compared with the placebo group: ^∗^*P* < .05. eGFR = estimated glomerular filtration rate, FZQZ = Fu-Zheng-Qu-Zhuo.

**Figure 3:**
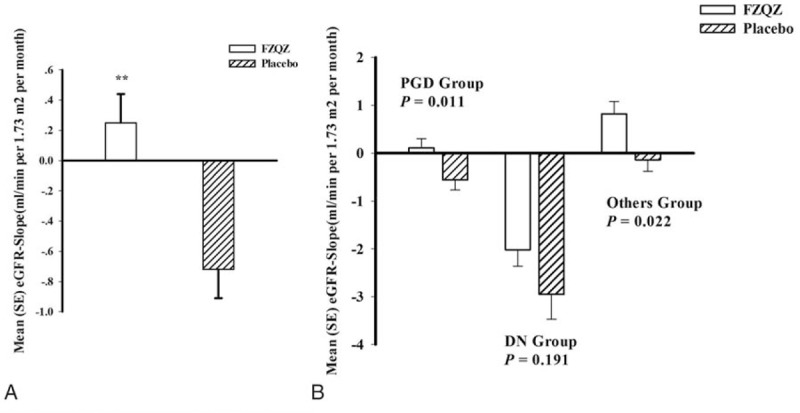
Analysis of the mean eGFR-Slope in the FZQZ and placebo groups in the in-trial phase. (A) Mean eGFR-Slope for the FZQZ and placebo groups; (B) CKD-associated subgroups analysis of mean eGFR-Slope in the FZQZ and placebo groups. Data presented as mean ± standard error. Compared with the placebo group: ^∗∗^*P* < .01. CKD = chronic kidney disease. DN = diabetic nephropathy, eGFR = estimated glomerular filtration rate, FZQZ = Fu-Zheng-Qu-Zhuo, Others = combination of the remaining CKD-associated subgroups, including tubulointerstitial disease, hypertension, ischemic nephropathy, autosomal polycystic kidney disease, and unknown, PGD = primary glomerular disease.

In the FZQZ group, eGFR increased from a baseline of 42.54 ± 10.88 to 48.22 ± 16.29 mL/min per 1.73 m^2^ by month 4 and gradually decreased to 45.55 ± 17.23 mL/min per 1.73 m^2^ at the end of the in-trial phase, whereas eGFR decreased progressively in the placebo group: 43.42 ± 11.91 to 35.94 ± 17.80 mL/min per 1.73 m^2^ (Fig. 2). The eGFR change from baseline was 3.52 ± 12.19 in the FZQZ group and −6.02 ± 13.36 mL/min per 1.73 m^2^ in the placebo group; the between-group difference 9.54 mL/min per 1.73 m^2^ (95% confidence interval [CI]: 4.15–14.93, *P* *=* .001). FZQZ was associated with a mean eGFR-Slope of 0.25 ± 1.44 versus −0.72 ± 1.46 mL/min per 1.73 m^2^ per month for the placebo group during the in-trial phase, with a between-group difference of 0.97 mL/min per 1.73 m^2^ per month (95% CI: 0.44–1.50, *P* < .001). It appeared that the treatment effect difference in terms of the eGFR-Slope in the FZQZ group was attributed to patients without DN (Fig. 3).

### 3.4 Secondary outcomes

**Figure 4:**
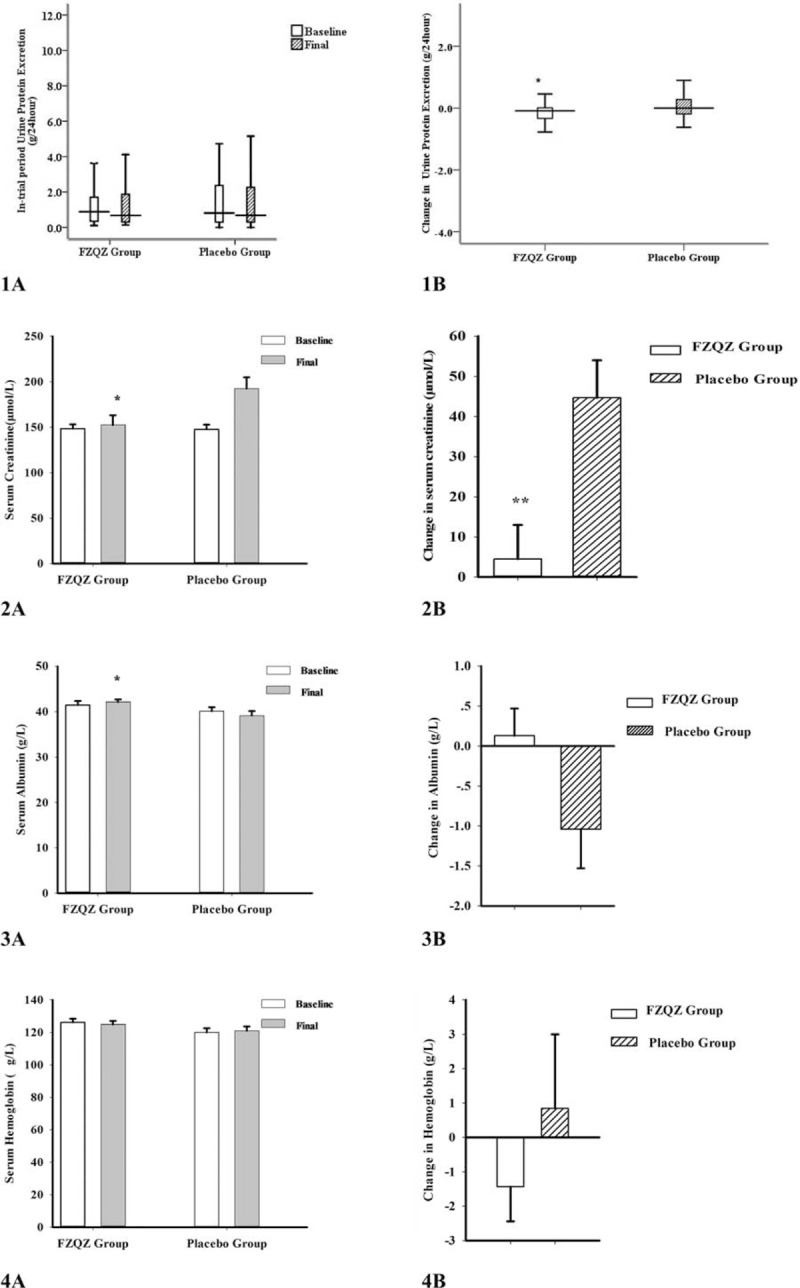
Change in therapeutic effect indices in the FZQZ and placebo groups in the in-trial phase. Column A: baseline and final levels of 24-h urinary protein excretion, serum creatinine, serum albumin, and hemoglobin. Column B: changes from baseline in levels of 24-h urinary protein excretion, serum creatinine, serum albumin, and hemoglobin. Data presented as mean ± standard error or median and 95% range. Compared with the placebo group: ^∗^*P* < .05, ^∗∗^*P* < .01. FZQZ = Fu-Zheng-Qu-Zhuo.

Baseline and final data of 24-h UP, SCr, ALB, and HGB were collected and analyzed as therapeutic effect indices (Fig. 4). Subgroup analyses according to the CKD-associated causes of these indices in the FZQZ and placebo groups were also conducted (Supplementary Table 3). Potassium and ALT were collected and analyzed as safety indices (Table 3). A significant mean difference in the SCr changes from baseline between the FZQZ and placebo groups was seen at the end of the in-trial phase (−40.20 μmol/L [95% CI: −65.32 to −15.07]), and the difference was mainly due to non-DN subgroups (Supplementary Table 3). The FZQZ group showed decreased 24-h UP, whereas it was shown to increase in the placebo group, with a between-group difference of 0.02 g (IQR: −0.23, 0.13, *P* *=* .049); the difference was mainly ascribed to the PGD subgroup (Supplementary Table 3). Though we were underpowered to detect a significant mean difference in ALB changes (1.17 g/L [95% CI: −0.02 to 2.36, *P* *=* .053]), the FZQZ group showed higher levels of final ALB than those of the placebo group (42.13 ± 4.28 vs. 39.14 ± 7.31 g/L [*P* *=* .010]). No significant differences in the changes in HGB values were observed.

**Table 3:**
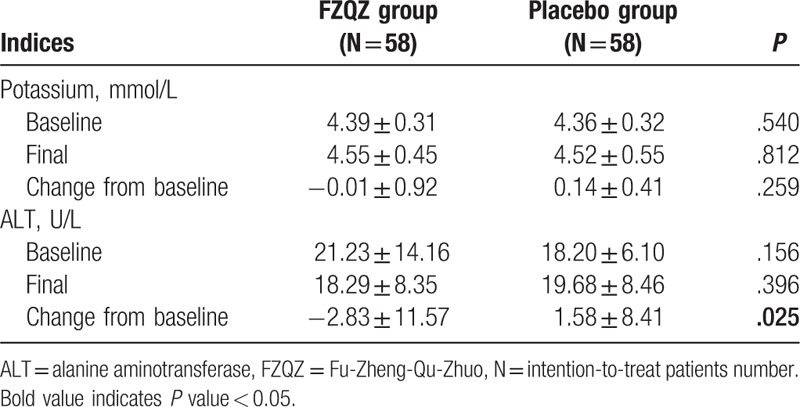
Baseline and final data of safety indices.

**Figure 5:**
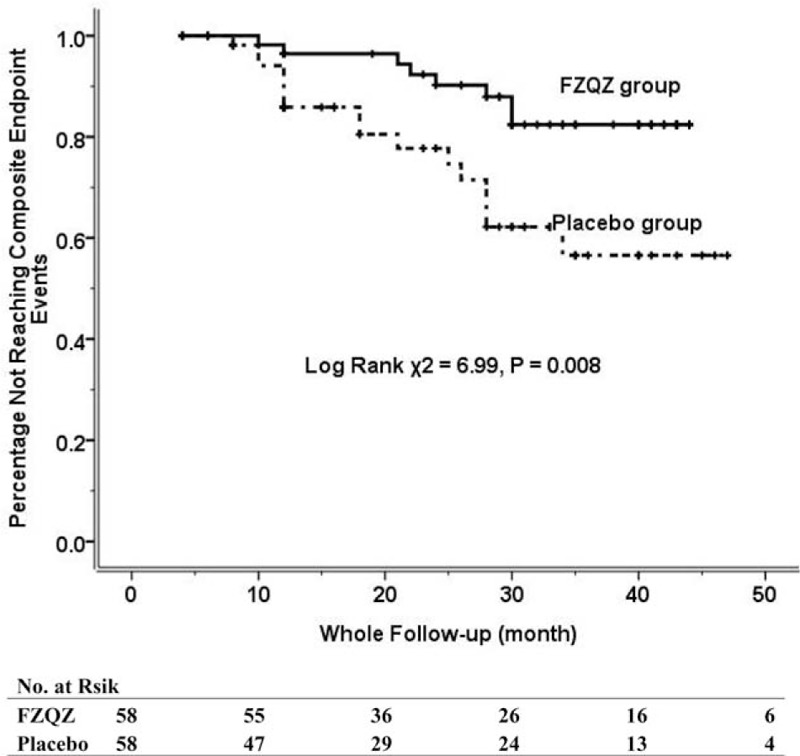
Kaplan–Meier curve for composite endpoint events throughout the entire follow-up period (in-trial and post-trial phases). Composite endpoint events were defined as the initiation of long-term dialysis, chronic kidney disease-related death, or the doubling of serum creatinine.

During the in-trial follow-up, 2 (3.44%) out of 58 patients in the FZQZ group showed doubled SCr, but without the need for renal replacement; 7 (12.07%) out of 58 patients in the placebo group reached a composite endpoint (1 initiation of long-term hemodialysis and 6 doubling of SCr). During the post-trial follow-up, another 6 patients in the FZQZ group reached a composite endpoint (1 initiation of long-term hemodialysis and 5 doubling of SCr), in total, 8 (13.79%) composite endpoint events happened in the FZQZ group; whereas another 9 patients in placebo group reached a composite endpoint (1 death, 1 initiation of hemodialysis, and 7 doubling of SCr); cumulatively, a total of 16 (27.59%) composite endpoint events occurred in the placebo group (Fig. 1). Although there was no significant difference in the occurrence of composite endpoint events between the FZQZ and placebo groups during the in-trial phase (3.44% vs. 12.07%, log rank χ^2^ = 3.73, *P* = .053), a significant difference was observed in the post-trial phase (13.79% vs. 27.59%, log rank χ^2^ = 8.04, *P* = .005) (Fig. 5). Multivariate Cox regression revealed that exposure to the FZQZ oral liquid was associated with a reduced risk of composite endpoint events during the entire follow-up period (HR = 0.42, 95% CI: 0.16–1.11, *P* = .038). Moreover, older age (HR = 0.95, 95% CI: 0.90–0.99, *P* = .024) and higher levels of HGB (HR = 0.96, 95% CI 0.90–1.00, *P* = .032) were predictors of better renal outcomes in this study (Table 4).

**Table 4:**
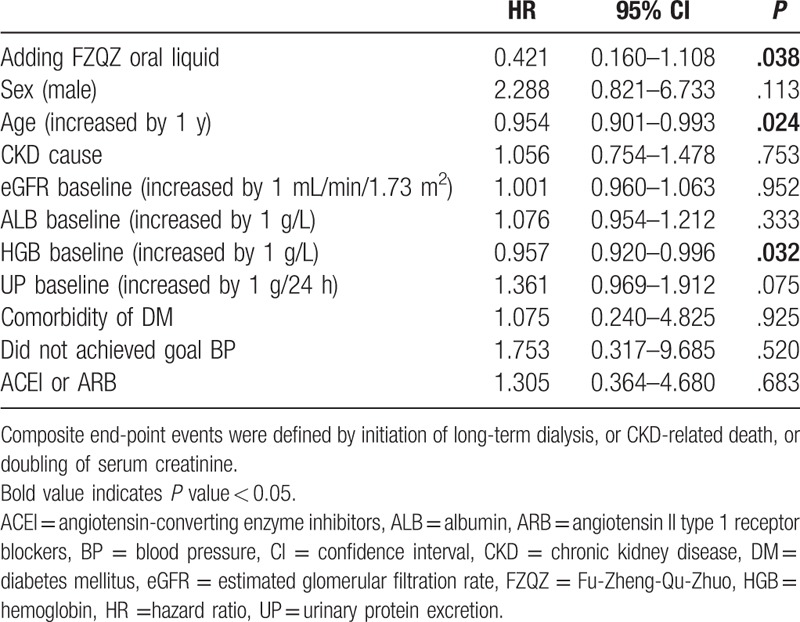
Risk factors for composite end-point events in whole follow-up.

### 3.5 Safety

Although the ALT changes from baseline were significantly different, −2.83 ± 11.57 or 1.58 ± 8.41 U/L for the FZQZ and placebo groups (*P* = .025), respectively, the ALT of most of the patients was in the normal range during the treatment phase (Table 3). There was no significant difference in other adverse events between the FZQZ and placebo group (Table 5). One death case was observed in the placebo group in the post-trial phase, and the cause of death was acute ST-segment-elevation myocardial infarction.

**Table 5:**
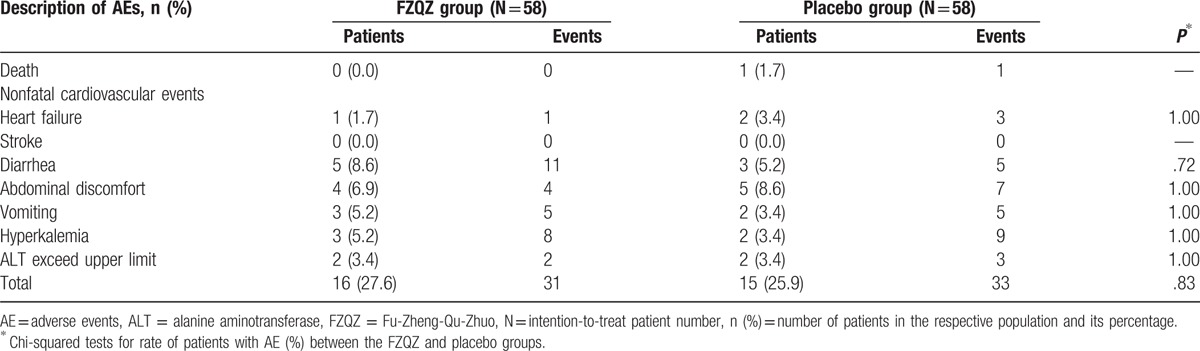
Summary of adverse events after randomization.

Patients reported that halitosis due to high blood urea nitrogen levels had decreased, along with easier bowel movements and softer stools, improved appetite, and increased physical function after using FZQZ.

## 4 Discussion

It has been estimated that the worldwide use of renal replacement therapy is projected to more than double to 5.439 million people by 2030, with the most growth happening in Asia, and the largest treatment gaps occur in low-income countries, particularly in Asia.^[13]^ In China, the overall prevalence of CKD is 10.8%, and the adjusted prevalence of eGFR <60 mL/min per 1.73 m^2^ is 1.7%^[2]^; therefore, the development of effective strategies to slow CKD progression is of considerable interest, especially in developing countries with huge populations such as China. Although many Chinese patients with CKD and some nephrologists deeply believe that TCM herbal medicine will bring benefits in CKD treatment in light of their experiences, the evidence from randomized clinical trials has been severely insufficient. In the present clinical trial, we tested the effect of the combined usage of a TCM herbal formula (FZQZ oral liquid) and standard integrated therapy on patients with CKD stage 3 or 4, and our results demonstrated that the usage of FZQZ significantly improved renal function as assessed by the eGFR-Slope. The risk of kidney failure was also reduced in the post-trial phase, though not in the in-trial phase.

Treatment with FZQZ was shown to decrease UP and showed some benefit effect in terms of ALB as well. Subgroup analysis according to CKD-cause indicated that the above-mentioned treatment effect difference between the FZQZ and the placebo was mainly attributed to patients without DN, especially patients with PGN. Urinary protein is a well-recognized independent risk factor of renal function decline^[14]^; therefore, the superior renal protection effect in the PGN subgroup might be associated with FZQZ-reduced UP, which was proven in our preliminary study.^[9]^

The components of FZQZ oral liquid are able to reinforce Qi, activate blood, dissolve dampness, and descend turbidity in patients with CKD. Alongside their well-established roles in TCM, the mechanism underlying the effects of some of these components has also been elucidated in a modern medicine context. For example, *Astragalus membranaceus* and *Angelica sinensis* have been shown to have antifibrotic effects in obstructive nephropathy, and the molecular mechanism may be related to increased degeneration of ECM, decreased reactive oxygen species reaction, and changes in calcium–phosphate metabolism.^[15]^ Moreover, *Alisma orientalis*, *Poria cocos*, and ginsenoside Rg1, the active component of ginseng, were reported to attenuate renal fibrosis in a unilateral ureteral obstruction rat model.^[16–18]^ Rhubarb and its derived components were shown to inhibit renal fibrosis and inflammation and to repress the deterioration of renal function.^[19,20]^ Another important pharmacological function of rhubarb in delaying the progression of CKD was associated with its cathartic effect. According to TCM theory, a common route of CKD pathogenesis is through the retention of toxins, and some components of the toxins commonly discharged from the kidney must be expelled through the colon in patients with CKD; therefore, a rhubarb-based compound has been applied to CKD treatment since the 1950s in China. Recent studies have revealed that the colon is an important organ in the generation of uremic toxins. Colon-derived toxins not only promote CKD progression, but are also closely linked with mortality in patients with CKD.^[21]^ Rhubarb-based compounds can help regulate intestine flora and reduce intestinally derived uremic toxins produced by gut bacteria, which provides the basis of a novel strategy for delaying CKD progression.^[22]^ In our study, the obvious increase in eGFR that happened quickly in the first 4 months of treatment in the FZQZ group might be associated with the improved bowel elimination of creatinine, whereas the gentle and stable increase in eGFR observed during the subsequent 8 months, along with the decreased risk of renal failure during the entire follow-up period in the FZQZ group, reveals its deeper effectiveness in delaying CKD progression. Meanwhile, improvements in appetite and bowel habits were reported from the patients treated with FZQZ, which, along with the trend toward increased ALB levels in that group, implies a gut-kidney axis that might be a mechanism of FZQZ's treatment effect. The role of the other herbal components in the FZQZ oral liquid in promoting renal protection requires further investigation.

Although the placebo oral liquid was similar to the FZQZ oral liquid in size, color, and shape, but lacked any active herbal medicine, the special smell and taste of the herbal medicine was difficult to imitate, and we were unable to rule out the possibility that such differences may have lead to participant unblinding and impacted compliance, and also resulted in the high dropout rate in the placebo group; therefore, all of the outcomes were analyzed according to ITT.

Due to a research grant limitation, renal function estimates (eGFR) derived from creatinine-based equations, noncreatinine-based GFR such as cystatin C measurement, or direct GFR measurements by radionuclide method were all absent. The study was also limited by a relatively small sample size and short treatment phase; hence, we merely defined eGFR-Slope, the surrogate end point, as primary outcome, kidney failure events’ analysis was provided as a secondary outcome.

In conclusion, our data demonstrated some beneficial effects of FZQZ in the attenuation of the progression of CKD stage 3 to 4. In addition, there were no apparent adverse events in the FZQZ group in the present study. This study may validate a new therapy option for patients with CKD with advanced kidney impairment. Since the sample size and follow-up period were limited, future larger-scale studies are needed to verify these findings.

## Acknowledgments

The authors would like to thank Jin-Wei Wang, PhD, from Peking University First Hospital for guidance in biostatistics in this study. The authors also would like to thank the following team members for their contributions to the success of this trial.

Research coordinators and Research Assistants (2010–2014): X-RR, X-WD, KP, LW, B-MH (Nephrology Department of Guang’anmen Hospital, China Academy of Chinese Medical Sciences), X-JW, Lv-Gui Fang (Nephrology Department of South Area of Guang’anmen Hospital, China Academy of Chinese Medical Sciences), N-NZ, L-XK (Nephrology Department of Beijing Fangshan Hospital of Traditional Chinese Medicine), and Tingting Ding (Drug clinical trial institution of State Food & Drug Administration of Guang’anmen Hospital, China Academy of Chinese Medical Sciences). The Research Coordinators and Research Assistants screened patients, obtained consent, randomized participants, and collected samples and data.

DSMB: Hai-Bo Yin and Jie Qiao (Research Ethics Committee of Guang’anmen Hospital). The Data and Safety Monitoring Board reviewed 2 formal interim analyses and regular reports of our primary composite outcome as well as serious adverse events.

OHRI statisticians: F-ML (Drug clinical trial institution of State Food & Drug Administration of Guang’anmen Hospital, China Academy of Chinese Medical Sciences). The statisticians performed quality assurance checks on data and conducted the study analysis.

OHRI data entry: Hai-Tao Lu, Xi-Yu Li, and Huan Guo (Beijing University of Chinese Medicine). Data entry personnel uploaded Case Report Forms, reconciled data queries, and conducted quality assurance checks on the database.

Data access and responsibility: The principal investigator (SL) had full access to all of the data in the study and takes responsibility for the integrity of the data and the accuracy of the data analysis.

## Supplementary Material

Supplemental Digital Content
