# Supplementary material for: Beneficial effects of Fu-Zheng-Qu-Zhuo oral liquid combined with standard integrated therapy in patients with chronic kidney disease (stage 3–4): A randomized placebo-controlled clinical trial
Source: Medicine (Baltimore). 2017 Jul 14;96(28):e7448. doi: 10.1097/MD.0000000000007448 (PMC5515754; doi:10.1097/MD.0000000000007448)
Supplement: Supplemental Digital Content [file medi-96-e7448-s001.doc]

**Supplementary information**

Integrated treatment protocol for all recruited patients with CKD in this trial:

The following parameters were controlled in all recruited patients with CKD: (1) Dietary protein was restricted to 0.6 g/(kg·d) (50% high quality protein) with sufficient calorie supply (30-35 kcal/kg·d); (2) High blood pressure was controlled by using anti-hypertensive agents (calcium-channel antagonists, diuretics, alpha- or beta-blockers, diuretics, or some combination of these medications) when necessary to achieve a systolic blood pressure (SBP) of less than 140 mmHg and a diastolic blood pressure (DBP) of less than 90 mmHg (for patients older than 70 with cardiovascular diseases, an SBP of less than 150 mmHg was acceptable). ACEI and ARB were excluded unless the patients had received them before being recruited, and the agent(s) and dosage were kept unchanged during the entire observation period; (3) Blood lipid levels were controlled by anti-hyperlipidemic agents, including atorvastatin and/or fenofibrate when necessary to achieve a low-density lipoprotein cholesterol (LDL-CH) level of less than 2.6 mmol/L and triglycerides (TG) less than 1.7 mmol/L; (4) Blood glucose was controlled by insulin or oral hypoglycemic agents when necessary to achieve a HbA1c of 6.5-8.0%; (5) Sodium bicarbonate was supplemented when necessary to achieve a serum HCO_3_^-^ of ≥ 22 mmol/L; and (6) Ferrous succinate, folic acid, and erythropoietin were supplemented when necessary to achieve an HB of 90-110 g/L.

**Supplementary Table 1**

Components of Fu-zheng-qu-zhuo oral liquid

| **Latin Binomial Name** | **English Name** | **Part Used** | | | **Type of Product** | | **Weight (g)** | |
| --- | --- | --- | --- | --- | --- | --- | --- | --- |
| Panax ginseng C. A. Mey. | Radix Ginseng | | Root | Raw (dry) | | 10 | |  |
| Astragalus membranaceus | Radix Astragali | | Root | Raw (dry) | | 15 | |  |
| *Angelica sinensis* | Radix Angelicae | | Root | Raw (dry) | | 12 | |  |
| Rheum .officinale baill | Radix Rhubarb | | Root | Raw (dry) | | 6 | |  |
| Plantago asiatica L. | Asiatic plantain herb | | Whole | Raw (dry) | | 12 | |  |
| *Poria cocos* | Sclerotium Poriae Cocos | | Sclerotium | Raw (dry) | | 12 | |  |
| Alisma orientalis (Sam.) Juzep. | Rhizoma Alismatis | | Tuber | Raw (dry) | | 12 | |  |
| Haematitum | Hematite | | Whole | Raw (dry) | | 20 | |  |
| Caulis Spatholobi | Suberect spatholobus stem | | Ratan | Raw (dry) | | 15 | |  |
| Herba Agastaches | Wrinkled Gianthyssop Herb | | whole | Raw (dry) | | 10 | |  |
| Perilla frutescens(L.) Britton | caulis perillae | | Stem | Raw (dry) | | 10 | |  |
| Radix Paeoniae Rubra | peony root | | Root | Raw (dry) | | 12 | |  |
| Radix Paeoniae Alba | Debark peony root | | Root | Raw (dry) | | 12 | |  |
| Ligusticum chuanxiong Hort. | Szechwan lovage rhizome | | Root | Raw (dry) | | 10 | |  |

**Supplementary Table 2**

**Study medicine** exposure and compliance rate in the in-trial phase

| Medicine Compliance | FZQZ group (N=58) | Placebo group (N=58) | *P* |
| --- | --- | --- | --- |
| Compliance rate (%) | 94.1±15.6 | 86.2±20.0 | **0.020** |
| Min, Max | 25.0, 100.0 | 20.0, 100.0 |  |
| **Categorical summary-n (%)** |  |  | **0.022** |
| ＜33% | 1(1.7) | 2(3.4) |  |
| ≥ 33% to< 66% | 2(3.4) | 5(8.6) |  |
| ≥ 67% to < 99% | 6(10.3) | 20(34.5) |  |
| ≥99% | 49(84.5) | 31(53.4) |  |
| **Duration of treatment (mth)- n (%)** |  |  | 0.105 |
| ＜4 | 1(1.7) | 2(3.4) |  |
| ≥4 to < 8 | 2(3.4) | 9(15.5) |  |
| ≥8 to < 12 | 1(1.7) | 2(3.4) |  |
| ≥12 | 54(93.1) | 45(77.6) |  |

Note and Abbreviation: Patients received a study medication kit every month that consisted of 10 bottles of study medicine (200 mL per bottle). To evaluate study medication compliance, patients were asked to return all bottles (used and unused). Site personnel counted the remaining liquid to calculate the compliance rate.

N = intention-to-treat patients number; n (%) = number of patients in the respective population and its percentage; Compliance rate = Total dose take/total dose required×100.

Duration of treatment (month) = [(date of last dose) – (date of first dose)] /30

**Supplementary Table 3**

**Baseline and final Data of** **therapeutic effect indexes (CKD-cause subgroups)**

| **Indexes** | CKD-cause subgroups (n/n) | FZQZ group  (N=58) | Placebo group (N=58) | *P* |
| --- | --- | --- | --- | --- |
| ***Urinary protein (g/24h)*** | |  |  |  |
| *Baseline* |  | 0.89(0.34, 1.64) | 0.80(0.33, 3.04) | 0.451 |
|  | PGD(23/27) | 1.04(0.69, 1.55) | 1.26(0.65, 2.85) | 0.435 |
|  | DN(6/8) | 3.71(1.96, 6.45) | 4.75(4.31, 6.00) | 0.282 |
|  | Others(29/23) | 0.39(0.16, 0.97) | 0.34(0.22, 0.70) | 0.527 |
| *Final* |  | 0.66(0.30, 1.52) | 0.92(0.43, 3.07) | 0.074 |
|  | PGD(23/27) | 0.68(0.42, 1.52) | 1.59(0.67, 3.14) | 0.067 |
|  | DN(6/8) | 3.19(2.46, 5.63) | 4.92(3.34, 5.62) | 0.295 |
|  | Others(29/23) | 0.62(0.31, 1.42) | 0.45(0.25, 0.54) | 0.670 |
| *Change from baseline* | | -0.08(-0.33, 0.01) | 0.01(-0.19, 0.31) | **0.049** |
|  | PGD(23/27) | -0.19(-0.35, 0.00) | 0.16(-0.12, 0.85) | **0.016** |
|  | DN(6/8) | -0.32(-1.26, 0.29) | -0.46(-1.19, 0.63) | 0.945 |
|  | Others(29/23) | 0.00(-0.01, 0.01) | -0.03(-0.17,0.09) | 0.876 |
| ***Scr(umol/L)*** |  | 148.00±36.65 | 147.88±38.91 | 0.987 |
| *Baseline* | PGD(23/27) | 144.34±29.63 | 156.30±48.38 | 0.308 |
|  | DN(6/8) | 181.17±47.19 | 157.62±25.52 | 0.251 |
|  | Others(29/23) | 144.03±37.19 | 134.61±25.35 | 0.304 |
| *Final* |  | 152.47±80.61 | 192.55±93.49 | **0.015** |
|  | PGD(23/27) | 143.04±54.50 | 206.67±100.29 | **0.007** |
|  | DN(6/8) | 285.00±154.42 | 308.75±129.14 | 0.759 |
|  | Others(29/23) | 132.53±48.86 | 148.26±54.51 | 0.279 |
| *Change from baseline* | | 4.48±64.81 | 44.67±71.61 | **0.002** |
|  | PGD(23/27) | -1.30±51.03 | 50.37±67.34 | **0.004** |
|  | DN(6/8) | 103.83±130.88 | 109.00±97.05 | 0.934 |
|  | Others(29/23) | -12.04±35.42 | 13.87±46.17 | **0.022** |
| ***ALB(g/L)*** |  | 41.47±6.84 | 40.12±6.42 | 0.283 |
| *Baseline* | PGD(23/27) | 41.32±4.18 | 40.39±4.83 | 0.482 |
|  | DN(6/8) | 36.05±5.44 | 30.93±8.68 | 0.230 |
|  | Others(29/23) | 43.65±2.39 | 43.09±3.67 | 0.506 |
| *Final* |  | 42.13±4.28 | 39.14±7.31 | **0.010** |
|  | GN(23/27) | 41.40±4.10 | 39.25±4.63 | 0.095 |
|  | DN(6/8) | 36.50±3.93 | 27.19±8.92 | **0.025** |
|  | Others(29/23) | 43.74±3.34 | 43.29±3.91 | 0.660 |
| *Change from baseline* | | 0.12±2.57 | -1.05±3.73 | 0.053 |
|  | PGD(23/27) | 0.09±2.20 | -1.14±3.80 | 0.186 |
|  | DN(6/8) | 0.45±2.08 | -3.74±2.34 | **0.005** |
|  | Others(29/23) | 0.09±3.01 | 0.04±3.68 | 0.959 |
| ***Hemoglobin(g/L)*** |  | 126.17±16.17 | 120.00±19.06 | 0.069 |
| *Baseline* | PGD(23/27) | 125.44±13.36 | 124.50±18.77 | 0.844 |
|  | DN(6/8) | 104.25±6.19 | 101.32±20.23 | 0.709 |
|  | Others(29/23) | 131.36±15.67 | 121.56±14.49 | **0.029** |
| *Final* |  | 124.81±17.13 | 121.02±19.76 | 0.284 |
|  | PGD(23/27) | 124.43±14.98 | 124.20±21.61 | 0.966 |
|  | DN(6/8) | 103.13±6.04 | 104.99±18.55 | 0.797 |
|  | Others(29/23) | 129.53±16.83 | 122.85±14.91 | 0.153 |
| *Change from baseline* | | -1.43±7.68 | 0.85±11.57 | 0.218 |
|  | PGD(23/27) | -1.01±4.86 | -0.30±11.96 | 0.781 |
|  | DN(6/8) | -1.12±3.47 | 4.51±13.47 | 0.342 |
|  | Others(29/23) | -1.82±9.89 | 1.29±11.56 | 0.311 |

Abbreviation and definition: N = intention-to-treat patients number; n/n = number of patients in FZQZ or placebo group respective population; PGD = primary glomerular disease; DN = Diabetic nephropathy; Others = combining the remained CKD-cause subgroups, including tubulointerstitial disease, Hypertension, Ischemic nephropathy, AKPD and Unknown; ALB = albumin; Scr = serum creatinine.
